# Zebrafish models for congenital disorders of glycosylation (CDG): a systematic review

**DOI:** 10.1186/s13023-025-04016-4

**Published:** 2025-09-24

**Authors:** N. Gandoy-Fieiras, M. I. Quiroga, L. Sánchez

**Affiliations:** 1 Department of Zoology, Genetics and Physical Anthropology, Faculty of Veterinary, Universidade de Santiago de Compostela, Lugo, 27002 Spain; 2 Department of Anatomy, Animal Production and Veterinary Clinical Sciences, Faculty of Veterinary, Universidade de Santiago de Compostela, Lugo, 27002 Spain

**Keywords:** Zebrafish, Congenital disorder of glycosylation, Animal model, Glycosylation, CDG, Disease model

## Abstract

Glycosylation is a post-translational modification of proteins that involves the addition of glycan groups and is essential for their proper functionality. This highly complex process affects 70% of all human proteins. Mutations in genes involved in glycosylation pathways can lead to a group of rare genetic syndromes known as Congenital Disorders of Glycosylation (CDG). One of the workflows applied to study human diseases includes animal models, such as the zebrafish. This systematic review aims to explore the utility of the zebrafish model in studying congenital disorders of glycosylation. For this purpose, searches were conducted in PubMed, Web of Science, and Scopus using terms related to congenital disorders of glycosylation and zebrafish, covering studies published up to November 2024. A total of 36 articles were identified based on the inclusion criteria. The results provide a comprehensive overview of these studies. The analysis reveals that CDGs related to N-glycosylation are the most frequently studied, morpholinos are the predominant technique used, zebrafish glycosylation genes exhibit a high degree of homology with human genes, and zebrafish models successfully replicate many of the clinical features observed in human CDG patients. This review highlights that zebrafish is a valuable strategy for studying CDG, offering important insights into the pathophysiology of these disorders and contributing to the development of potential therapeutic approaches.

**Supplementary Information:**

The online version contains supplementary material available at 10.1186/s13023-025-04016-4.

## Background

Congenital disorders of glycosylation (CDG) have been described for first time in 1980 by Jaak Jaeken [1]. Since then, the number candidate genes and new types of CDG grow exponentially. CDG is as a rare syndrome that includes more than 150 different diseases [2]. Although all patients have defects in the glycosylation process, the great complexity of this process leads to a wide heterogeneity of symptoms among patients, making it very difficult to establish a specific phenotype for CDG, or even for each subtype [3].

Glycosylation is defined as a post-translational modification by the addition of glycan groups to a protein or lipid. This process occurs in 70% of the proteins in eukaryotic cells and will determine the correct functionality of the proteins. It is a biochemical mechanism that is highly conserved in all species, due to the functional importance that glycans confer on proteins or lipids [4]. These functions include protecting proteins from denaturation or proteases, serving as recognition targets, interfering with correct protein folding, promoting cell communication, among others [5]. Glycosylation plays a fundamental role in the health of organisms, which can be altered in common diseases such as diabetes, cancer or inflammatory diseases [6]. Consequently, the study of different sub-types of CDG generate knowledge for other disease and physiological process.

### Zebrafish as a model for disease

Zebrafish is a very usefully animal model in biomedicine research, there are zebrafish models for the majority of disease: development, metabolic [7], cardiac [8], neurological [9], behavior, and, indeed, genetics [10].

The use of zebrafish as an animal model dates to the 1980s, when George Streisinger became interested in zebrafish as a potential vertebrate model and publish his first article in 1981 in the Nature [11] titled “Production of clones of homozygous diploid zebra fish (*Brachydanio rerio*)”. Since then, zebrafish have demonstrated numerous advantages as an animal model, external fertilization and the transparency of embryos during the first days of life, which allows direct visualization of organ morphogenesis and the study of development in vivo, well known since 1995, thanks to Kimmel’s work [12].

Moreover, the use of zebrafish grew exponentially, especially from 2013 onwards when its genome was fully sequenced [13]. The analyse of genome shows the great the high similarities between human and zebrafish genetic material. It has orthologs for 76–82% of human genes involved in diseases. Therefore, this organism is extensively used in research of genetic diseases.

This review, integrating all the previous information, is presented as a response to the following scientific question: *“What zebrafish models have been developed to study congenital disorders of glycosylation*,* and how do their findings compare to the clinical manifestations observed in human patients?”*

## Materials and methods

Systematic review methodology was chosen for explore the state of art in the use of the zebrafish model for congenital disorders of glycosylation. For this purpose, keywords related to the selected topic were identified, and the search platforms PubMed, Web of Science, and Scopus were used. The search algorithm was designed using terms related to congenital disorders of glycosylation and zebrafish, along with their thesaurus (show in Additional File 1). The date of the last search is 28th November 2024.

The search results were filtered using the available filters in the selected databases to exclude congress proceedings, awards, and other non-research works. Only peer-reviewed articles were retained for analysis. The inclusion criteria were defined as: (a) articles reporting results on zebrafish models with mutations in genes related to glycosylation [14]. The exclusion criteria included: (a) models for other diseases that explore glycosylation patterns (e.g., cancer, diabetes, etc.), and (b) review articles. In addition, studies were manually added to ensure a more comprehensive coverage of the existing literature. These studies meet the inclusion criteria and were identified through active searches for genes related to glycosylation, references cited in the reviewed articles, or participation in conferences specifically focused on this topic.

Supplementary information regarding patient symptoms and protein functions was obtained from publicly available databases such as UniProt, OMIM, Orphanet, and GeneCards, as well as from case report articles when available. These additional resources provided valuable insights, ensuring a more comprehensive understanding of the clinical and molecular aspects relevant to the study.

The different articles were classified based on the glycosylation process in which the implicated gene participates. To achieve this, various databases were consulted, with the web resource CDG Hub being ultimately selected due to its comprehensiveness.

The bibliometric analysis was performed using Bibliometrix and Biblioshiny R-tool [15], while the figures were generated using Microsoft Excel. This review adopts the PRISMA (Preferred Reporting Items for Systematic Reviews and Meta-Analyses) methodology. This framework ensures a transparent approach for identifying, selecting, and synthesizing relevant literature.

## Results

This review summarizes the key findings on the use of zebrafish as a model for studying Congenital Disorders of Glycosylation, highlighting the main discoveries and insights obtained from these studies.

**Fig. 1:**
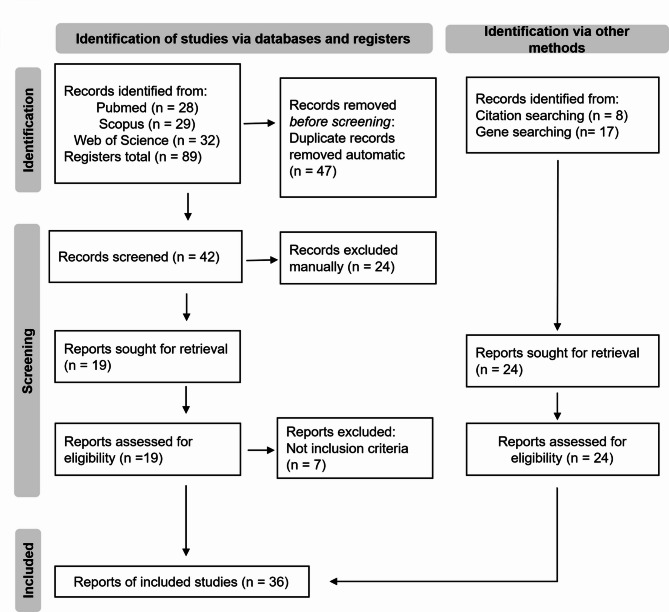
PRISMA 2020 flow diagram for new systematic reviews which included searches of databases, registers and other sources

The database search yielded a total of 89 articles. Following the removal of duplicates, abstract screening, and assessment for relevance to the topic, 12 articles were selected for inclusion. To complete this review and provide more detailed information on the topic, 24 additional articles were incorporated into the analysis. All of them met the inclusion criteria and were deemed relevant to the scope of this review. These were identified by specifically searching for the gene names of interest, references cited in the reviewed articles, or participation in conferences specifically focused on this topic. The final number of reports included in this review was 36 articles, an overview of the included and excluded papers are on Fig. 1. An extended summary of all included studies is presented in the Additional file 1.

**Fig. 2:**
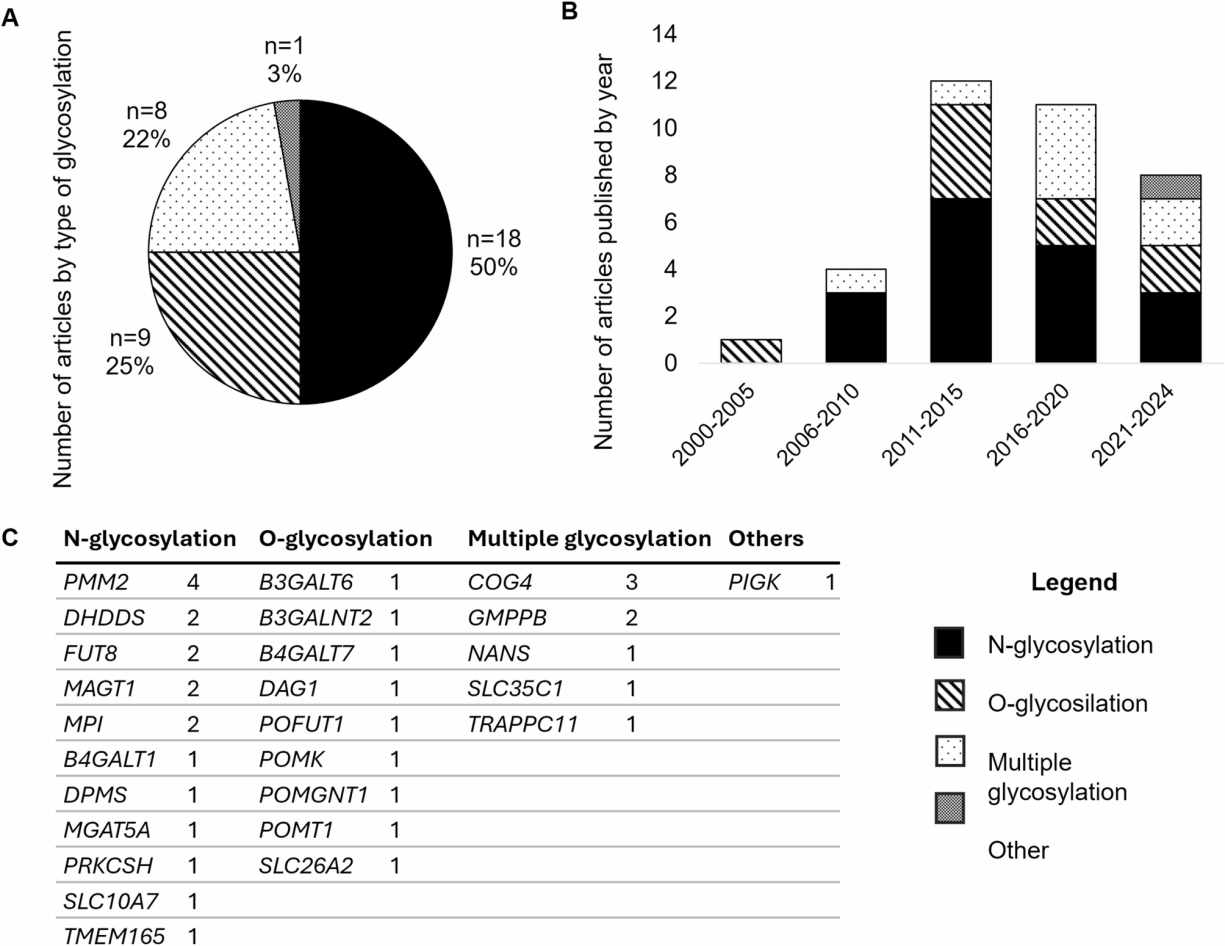
(A) Number of articles about zebrafish models for each type of glycosylation. (B) Number of articles published by year. (C) Number of articles associated with each gene categorized by glycosylation type

Following the classification criteria described above, it was observed that the most studied type of glycosylation in the literature reviewed is N-glycosylation (50%; *n* = 18), followed by O-glycosylation (25%; *n* = 9) and proteins involved in multiple types of glycosylation (22%; *n* = 8). There is one study classify in others (3%; *n* = 1) (Fig. 2A). The most studied genes are *PMM2* and *COG4*, with four and three articles, respectively. These are followed by *DHDDS*, *FUT8*, *MAGT1* and *GMPPB*, with two articles each. The remaining genes analyzed are represented by only one study per gene (Fig. 2C). The classification over the years shows that research using zebrafish models to study CDG began to be published in the 2000s, with a notable increase in the number of articles published between 2011 and 2015, potentially linked to the zebrafish genome sequencing in 2013 [13]. Publications on sub-types of CDGs are distributed across the years, with no clear trend showing more or less interest in specific types of glycosylation (Fig. 2B).

**Fig. 3:**
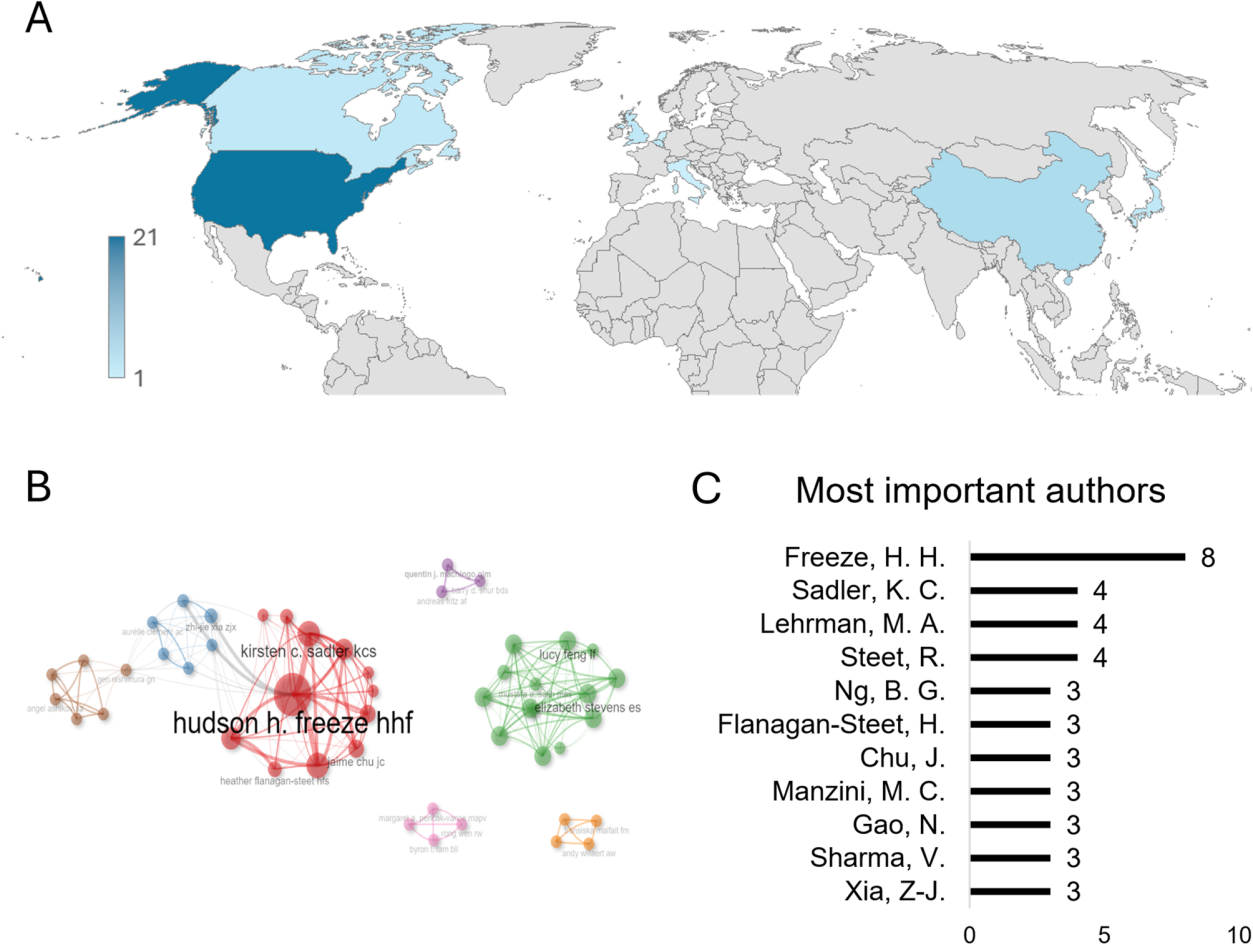
(A) Distribution of articles included in this review distributed by country in global world. (B) Principal authors of zebrafish models for CDG classify by clusters. (C) List of the most prolific authors and the number of papers for each one

Regarding the geographical distribution of the studies, 66% of the publications originate from North America, with the majority from the United States (60%; *n* = 21) and a smaller proportion from Canada (6%; *n* = 2). In Asia, 17% of the studies are concentrated, primarily in China (11%; *n* = 4) and Japan (6%; *n* = 2). Europe accounts for 17% of the scientific output, distributed across the United Kingdom (6%; *n* = 2), Belgium (6%; *n* = 2), Italy (3%; *n* = 1) and The Netherlands (3%; *n* = 1). This distribution was based on the affiliations of researchers contributing to zebrafish experiments, as their work represents the aim of this review (Fig. 3A).

The analysis of the authors and their relationships shows that the authors are distributed across seven clusters, with four clusters remaining independent and unconnected, while three clusters exhibit some degree of collaboration (Fig. 3B). Hudson H. Freeze stands out as the most prolific author, contributing as a co-author to eight papers. Following him, Kirsten Sadler, Mark Lehrman, and Richard Steet are among the most frequently featured authors in this field with 4 papers each one (Fig. 3c). More than 400 authors contributed to papers related to zebrafish models for CDG. During the review of the papers, it was noted that 12 of them not only included zebrafish models but also described the clinical features of patients or reviewed the most common mutations and primary symptoms. The authorship lists for these types of studies are typically very extensive, as they include contributions from both clinicians and researchers.

**Fig. 4:**
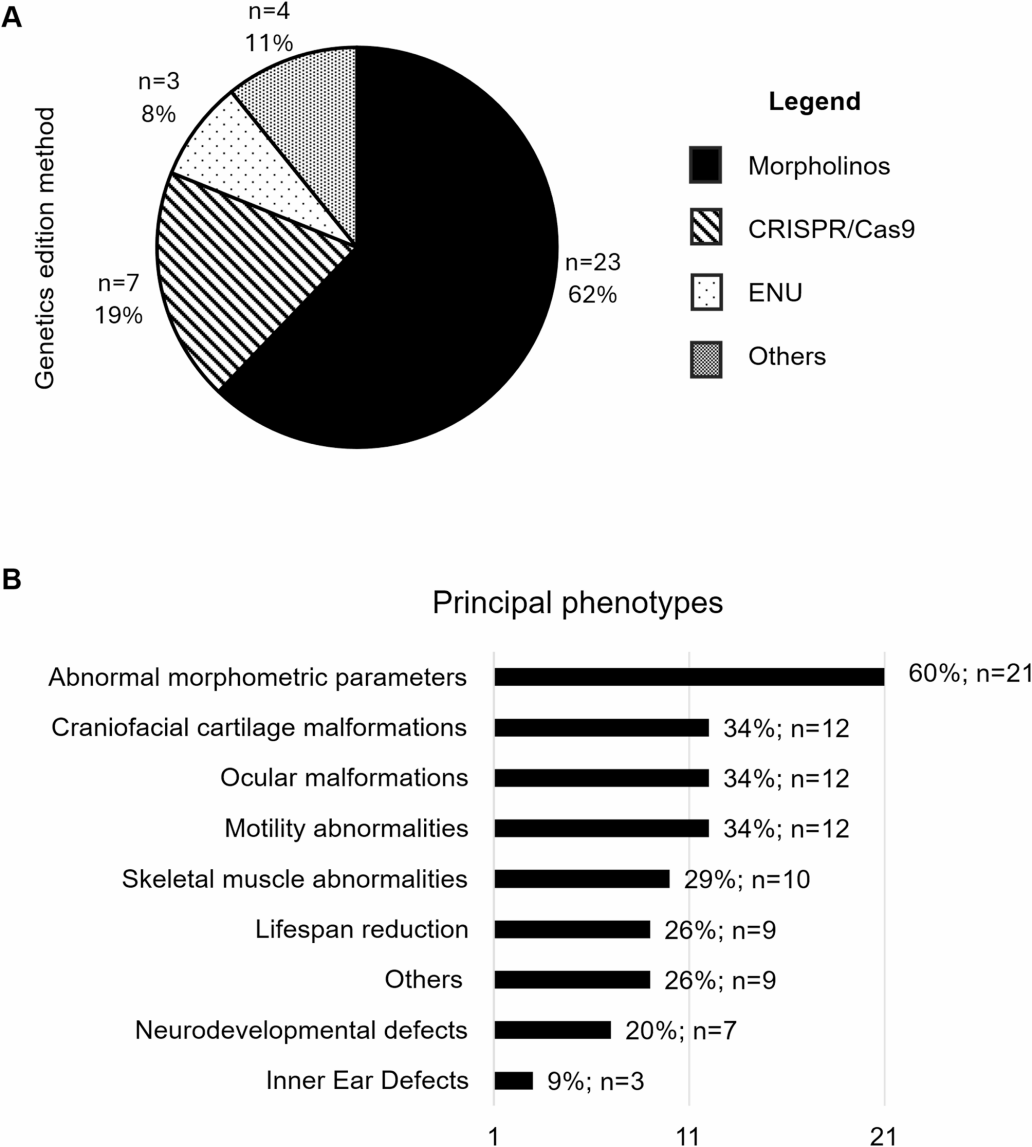
(A) Different genetics’ method used for creating zebrafish model to study glycosylation. (B) Overview of the principal phenotypes observed in zebrafish models and its percentage of appear

In terms of genetics, glycosylation-related genes exhibit a high degree of homology between the zebrafish genome and the human genome, with percentages ranging from 52.7 to 92.3%. This makes zebrafish an excellent candidate for the application of genome-editing techniques. The most widely used method is morpholinos (62%; *n* = 23), in second place is CRISPR/Cas9 (19%; *n* = 7), there are also studies using N-ethyl-N-nitrosourea (ENU) (8%; *n* = 3) and other techniques as employ human mRNA injections, (11%; *n* = 4) (Fig. 4A).

Zebrafish model phenotypes described include defects or abnormalities in multiple organs and systems (Fig. 4B). The most common phenotype observed is abnormal morphometric parameters (*n* = 21; 60%), such as short body length, microphthalmia, or shorter fins. This is followed by craniofacial cartilage malformations (*n* = 12; 34%). Sensory defects are also a recurrent phenotype, including ocular malformations (*n* = 12; 34%), often characterized by a lack of photoreceptors, and inner ear defects (*n* = 3; 9%).

In addition, zebrafish models frequently exhibit a short lifespan or high mortality rates within the first hour’s post-fertilization (*n* = 9; 26%). Another significant phenotype observed is motility impairment and erratic swimming behavior (*n* = 12; 34%), which is often associated with skeletal muscle defects, such as sparse muscle fibers (*n* = 10; 29%), and neurodevelopmental abnormalities, including shortened motor neuron axons (*n* = 7; 20%). Other phenotypes, such as liver steatosis, alterations in heart rate, abnormal melanin distribution and others have been observed in some models.

Efforts to rescue these phenotypes have focused on exploring different therapeutic strategies. A total of 8 models have explored the use of drugs or supplementation, three utilized sugar supplementations, while the others tested different types of drugs.

## Discussion

The aim of the present systematic review is to explore the state of the art regarding the application of zebrafish as a model for studying Congenital Disorders of Glycosylation and the results derived from these studies.

One of the challenges in CDG is the lack of cohesion in the nomenclature. Despite efforts to establish a standardized nomenclature for CDG [16, 17], glycosylation remains a broad concept, and some papers do not explicitly mention CDG. Instead, they explore what happens when specific genes fail, which results in the loss of valuable information directly related to the disease. This information can only be identified if the specific gene name is mentioned because they do not directly address CDG.

The classification of genes associated with CDG can vary depending on the source of information. For example, some sources include genes that do not directly participate in glycosylation but act as transporters, facilitating the passage of ions essential for glycosylation enzymes, as part of the CDG spectrum [14]. In contrast, other sources did not include these genes from the classification. Additionally, certain enzymes with unclear roles in the glycosylation process remain poorly categorized, reflecting gaps in our understanding and standardization of the field. An example of this is the dystroglycanopathies, a specific group of glycosylation defects that exclusively affect α-dystroglycan, an important component of skeletal muscle. As a result, this type of disease is sometimes not classified as a CDG properly [18].

Although attempts have been made to standardize nomenclatures, establish international organizations such as the World CDG Organization, and support initiatives like the International Rare Diseases Research Consortium (IRDiRC) to promote collaboration [19, 20], the inherent characteristics of these diseases—such as their low prevalence and the global dispersion of patients—limit the effectiveness of these efforts in practice. As observed among zebrafish researchers focused on CDG, small, isolated clusters of collaboration tend to form, with only limited interactions, primarily involving the most experienced authors in the field.

N-glycosylation seems the most studied, 50% of the zebrafish models found, this data contrast with the data of the most diagnosed CDG, in 2022 there are 33 subtypes of CDG related with N-glycosylation and 44 related with O-glycosylation [2]. Until now, N-glycosylation was the most studied process, with the enzymes involved and glycan binding sites being well understood. However, thanks to new omics techniques, knowledge of the O-glycosylation process has expanded, revealing that this process may be more complex than previously thought [21]. Another reason for the low number of diseases associated with O-glycosylation could be the naming of these syndromes. As previously mentioned, these diseases are often classified as dystroglycanopathies or given their own specific names, making it difficult for them to appear clearly in bibliographic searches [22].

Zebrafish is an excellent candidate for the application of genome-editing techniques due to their biological characteristics. Most of the zebrafish models used to study CDG employ morpholinos as a genetic editing strategy. These models allow researchers to obtain quick results by transiently blocking the candidate gene to check if the absence of that gene recapitulates the symptoms and signs observed in human patients [23]. Offering a very good opportunity to conduct a screening or initial assessment of the model. However, stable models can provide more detailed insights into disease progression over time and allows transmission of modifications to offspring. These models can be generated by classical methods as ENU mutagenesis, a potent, non-directed mutagenic method typically available in sperm libraries, such as those at ZIRC or by CRISPR/Cas9 [24], a directed and stable genetic editing method. In recent years, it has become the most popular choice for zebrafish researchers due to its ease of application in this specie. Combining these approaches offer valuable tools for understanding the mechanisms of the disease and makes the zebrafish an excellent model for searching for new therapeutic targets [25].

To conclude this state-of-the-art review, the following section will discuss all subtypes, comparing the most significant symptoms observed in patients with the primary phenotypes of zebrafish models for each gene. The Table 1 highlights the similarities between clinical manifestations in humans and experimental findings in zebrafish, demonstrating the utility of the model in studying disease mechanisms. This overview, it will provide a comprehensive overview of the utility of zebrafish as a model for studying CDG.

**Table 1:** Overview of the main symptoms observed in CDG patients, including the associated genes, and their corresponding phenotypes in zebrafish models. In bold the genes that share similar phenotypes in human and zebrafish

Human symptons	Zebrafish phenotype
Skeletal malformation	Abnormal morphometric parameters
***B3GALT6***,*** B4GALT1***,*** FUT8***,*** NANS***,*** SLC10A7***,* SLC26A2*,*** TMEM165***	*b3galnt2*,*** b3galt6***,*** b4galt1***,* b4galt7*,* cog4*,* dag1*,* dpms*,*** fut8***,* gmppb*,* magt1*,* mpi*,*** nans***,* pigk*,* pomk*,* pomt1*,* prkcsh*,*** slc10a7***,* slc35c1*,*** tmem165***
Dysmorphic features	Craniofacial cartilage malformations
***B3GALT6***,*** COG4***,*** MAGT1***,* MAGT5*,*** MPI***,*** PIGK***,*** PMM2***,*** SLC10A7***,*** TMEM165***	***b3galt6***,* b4galt7*,*** cog4***,* fut8*,*** magt1***,*** mpi***,* nans*,*** pigk***,*** pmm2***,*** slc10a7***,*** tmem165***
Loss of vision	Ocular malformations
***B3GALNT2***,*** DHDDS***,*** FUT8***,* GMDS*,*** GMPPB***,*** POMGNT1***,*** POMK***	***b3galnt2***,* cog4*,*** dhdds***,* fut8*,*** gmppb***,* mpi*,* pigk*,*** pomgnt1***,*** pomk***,* pomt1*,* slc10a7*
Reduced life expectancy	Lifespan reduction
-	*dpms*,* magt1*,* mpi*,* pmm2*,* pomk*,* pomt1*
Psychomotor retardation	Motility abnormalities
*B4GALT1*,* COG4*,*** DPMS***,* FUT8*,* MAGT5*,* MPI*,* PIGK*,*** PMM2***	*b3galnt2*,* b3galt6*,* dag1*,*** dpms***,* gmppb*,* magt1*,* pomk*,* pomt1*,*** pmm2***,* slc26a2*
Muscle weakness and hypotonia	Skeletal muscle abnormalities
***B3GALT6***,*** B4GALT7***,* COG4*,*** FUT8***,*** GMPPB***,* GMDS*,* MGAT5*,* MPI*,* NANS*,* PIGK*,* PMM2*,* SLC10A7*,* TMEM165*	***b3galt6***,*** b4galt7***,* dag1*,* dpms*,*** fut8***,*** gmppb***,* magt1*,* pomk*,* pomt1*
Ataxias, seizures, and brain abnormalities	Neurodevelopmental defects
*B3GALNT2*,* B4GALT1*,* COG4*,* DAG1*,*** GMPPB***,* MGAT5*,*** MPI***,*** PIGK***,* POMK*,* POMT1*,* POMGNT1*,*** PMM2***,* TRAPC11*	*fut8*,*** gmppb***,*** mpi***,*** pigk***,*** pmm2***,* slc35c1*
Sensorineural hearing loss	Inner ear defects
*GMPPB*,* SLC10A7*	*cog4*,* slc26a2*
Others	Others
***PRCKSH***,* TMEM165*	*gmppb*,* magt1*,* magt5a*,* mpi*,* pmm2*,* pofut1*,*** prkcsh***,* trappc11*

### Zebrafish models for N-glycosylation disorders

#### DHDDS-CDG

Dehydrodolichyl diphosphate synthase is involved in the synthesis of dolichol phosphate. Dolichol serves as the anchor for glycans during the glycosylation process. Mutations in this gene are related with retinitis pigmentosa in humans. In a zebrafish morpholino model, researchers found that the embryos were unable to respond to light and dark stimuli. Histological studies revealed that the photoreceptors in the eyes of morpholino-treated embryos were either shorter or completely absent [26]. Electron microscopy of zebrafish revealed significant retinal abnormalities, including the near absence of photoreceptor outer segments and mitochondrial defects. These findings suggest that early mitochondrial dysfunction may contribute to vision problems, linking *DHDDS* mutations to both retinal and membrane dysfunction [27].

#### PMM2-CDG (also known as CDG-Ia)

One of the most common sub-type of CDG, is caused by mutations in the *PMM2* gene, which encodes the enzyme phosphomannomutase 2 and affects one of the early steps in N-glycosylation, catalyzing the conversion of mannose-6-phosphate to mannose-1-phosphate. There are two zebrafish models: a morpholino model [28] and an ENU model [29]. In both models, researchers have described alterations in craniofacial cartilage development, specifically in the principal structures of Meckel’s cartilage and the ceratohyal, at 4 days post-fertilization (dpf) and 6 dpf, respectively. The observed alterations in cartilage development in zebrafish models of PMM2-CDG correlate with the facial phenotypes described in patients. These findings underscore the relevance of facial dysmorphology as a key aspect in diagnosing PMM2-CDG and assessing its severity [30]. Additionally, the zebrafish cannot live more than 2 weeks post fertilization (wpf). PMM2-CDG patients have a mortality rate of 20% within the first few years of life [31] with 30% of these deaths attributed to cardiac problems [32].

#### FUT8-CDG

α-1,6-Fucosyltransferase is an enzyme involved in determining the terminal structure of glycans and catalyzes the addition of fucose to the core structure of N-glycans. In zebrafish models, *fut8* morphants exhibit abnormal skeletal muscle structure, including defects in myosepta and sarcomere organization [33]. Additional phenotypes observed in other morpholino models include smaller eyes, small forebrains, a curved body axis, and disorganized motor neurons. Moreover, multiple cell types in the retina were absent in *fut8* morphants [34]. Some of these phenotypes are described in patients, they present symptoms like poor growth, failure to thrive, hypotonia, skeletal anomalies, and delayed psychomotor development with intellectual disability.

#### MAGT1-CDG

Magnesium Transporter 1 is a component of the N-oligosaccharyl transferase, responsible for converting oligomannose N-glycans into hybrid and complex N-glycans. Morpholino and CRISPR/cas9 models in zebrafish showed reduced embryo survival, notable malformations, and decreased hatching rates. Mutants exhibited impaired touch-evoked escape responses, with less than 40% of *mgat1b*−/− mutants responding immediately. Additionally, there was a decrease in swimming distance in response to vibration stimuli and an overall reduction in swimming distance during larval development. Light intensity in birefringence assays, used to study muscle structure, was lower in mutant embryos and larval zebrafish [35]. In humans, intellectual and developmental disability [36] and impaired immune-cell function, making patients susceptible to Epstein-Barr virus infections and other immune-related issues [37].

#### B4GALT1-CDG

Beta-1,4-galactosyltransferase 1 enzyme is involved in the biosynthesis of various glycoconjugates and saccharide structures. This disorder results in under-glycosylated serum glycoproteins and coagulation anomalies, leading to clinical features such as nervous system defects, psychomotor retardation, immunodeficiency, macrocephaly, and Dandy-Walker malformation [38]. Knockdown experiments using morpholinos show that this gene is essential for proper convergent extension movements during embryonic gastrulation. Its inhibition causes significant malformations in embryos due to impaired cell migration during the early developmental stages [39].

#### DPMS-CDG (CDG-Ie)

The Dolichol-phosphate mannosyltransferase complex, consisting of DPM1, DPM2, and DPM3, is responsible for transferring mannose from GDP-mannose to dolichol monophosphate, forming dolichol phosphate mannose. This is an ultra-rare type of CDG with less than 20 cases in literature. A morpholino model targeting this complex showed high embryonic mortality rates ranging from 25 to 50% within the first few days of life. Phenotypically, dystrophic skeletal muscle was observed, varying for each gene, along with apoptosis in embryos at 96 h post-fertilization (hpf). Behavioral analysis also demonstrated decreased spontaneous movement and a reduced response to touch in the morphants [40].

#### MGAT5A-CDG

N-acetylglucosamine transferase 5 A catalyzes the addition of N-acetylglucosamine (GlcNAc) in a beta 1–6 linkage to the alpha-linked mannose of biantennary N-linked oligosaccharides. The disease results in under-glycosylated serum glycoproteins, leading to a bleeding tendency and manifesting with defects in nervous system development, psychomotor retardation, dysmorphic features, hypotonia, coagulation disorders, and immunodeficiency. A stable zebrafish model created using CRISPR/Cas9 shows that inhibition of the N-glycosylation pathway enhances the regeneration of the lateral line and caudal fins [41].

#### MPI-CDG (also known as CDG-Ib)

MPI-CDG is a very rare variant of CDG, with only 35 cases described in the world [42]. It is involved in the formation of the glycan precursor by converting fructose-6-phosphate into mannose-6-phosphate. There is a zebrafish model using morpholinos. Researchers observed that 50% embryo lethality in the first 4 dpf [43], patients have a 23.5% of mortality all of them in infancy and early childhood [42]. Zebrafish embryos exhibited multi-systemic abnormalities such as small eyes, dysmorphic jaws, pericardial edema, a small liver, and curled tails. Moreover, a therapy based on mannose supplementation was tested and successfully rescued the phenotype of morpholino-treated fish [43].

#### PRKCSH-CDG (also known as CDG-Ih)

Beta-subunit of glucosidase II is involved in removing alpha-1,3-linked glucose residues from the Glc2Man9GlcNAc2 oligosaccharide precursor of immature glycoproteins. This CDG is characterized by a liver phenotype, with patients exhibiting multiple cysts in the liver [44], caused by the overgrowth of biliary epithelium and supportive connective tissue. This phenotype has been well described in a zebrafish morpholino model. In H&E-stained zebrafish, multiple cysts of varying shapes and sizes were observed in the livers of 4 dpf. Furthermore, this study tested the use of Pasireotide, which demonstrated the ability to inhibit hepatic cystogenesis [45].

#### SLC10A7-CDG

Solute Carrier Family 10 Member 7 is involved in the regulation of calcium and manganese homeostasis in the endoplasmic reticulum, which is essential for the proper function of enzymes. The most notable symptoms of this CDG include skeletal dysplasia, severe scoliosis, defective tooth formation, and other features such as facial dysmorphism, hearing impairment, and mildly impaired intellectual development [46]. A zebrafish model created using the morpholino strategy exhibits a severe phenotype, including whole-body edema, reduced head and eye size, and a curled body. Additionally, the morphants show significant craniofacial cartilage reduction or absence of structures and exhibit a marked reduction in the mineralization of major skeletal structures or their complete absence [47] .

#### TMEN165-CDG (also known as CDG-IIk)

Protein transmembrane 165 is involved in ion homeostasis, affecting glycan processing in the Golgi and lysosomal/endosomal function. Morpholino model of zebrafish show developmental abnormalities, including craniofacial defects at 4 dpf (affecting Meckel’s, ceratohyal, and palatoquadrate cartilages), shorter body length, and issues with cartilage and bone development. Notably, mRNA containing a patient mutation fails to rescue these cartilage defects in morphant embryos [48]. Patients with TMEM165-CDG commonly present with growth and skeletal abnormalities, such as growth retardation, bone dysplasia, craniofacial malformations, and facial dysmorphism. Neuromuscular symptoms include psychomotor delay, muscle weakness, and joint laxity. Cardiac and renal complications are also observed [49].

### Zebrafish models for O-glycosylation disorders

This type of disorders usually has another name or are classify as congenital muscular dystrophy (CMD) or dystroglycanopathy, due to the aberrant O-mannosylation of α-distroglycan [50].

#### Ehlers-Danlos syndrome (mutation in genes B3GALT6 and B4GALT7)

These enzymes are required for the biosynthesis of the tetrasaccharide linkage region of proteoglycans. Ehlers-Danlos syndrome characterized by an aged appearance, developmental delay, short stature, craniofacial disproportion, generalized osteopenia, defective wound healing, hypermobile joints, hypotonic muscles, and loose but elastic skin [51]. The zebrafish models successfully recapitulated the phenotypes observed in humans. These models exhibited external malformations characterized by a shortened body, microcephaly, a malformed posterior edge of the opercular apparatus, and bowed pectoral fins. These phenotypes result from the absence or profound delay in the development of cartilage elements and delayed mineralization across all regions of the head. Additionally, vertebral malformations were observed. Moreover, ultrastructural analysis revealed muscle defects, including disrupted actin patterns. Regarding glycosylation patterns, the total amount of sulfated glycosaminoglycans was significantly reduced [52, 53].

#### B3GALNT2-CMD

This gene transfers N-acetyl galactosamine in a β-1,3 linkage to N-acetyl glucosamine. B3GALNT2-CMD is characterized by visual symptoms, including ocular involvement in ten cases, which features optic nerve hypoplasia, microphthalmia, and blindness. Some patients also present neurological symptoms, such as ataxia or balance problems [54]. These symptoms are well recapitulated in morpholino models, which show mild retinal degeneration and severely impaired motility. The muscle phenotype displays reduced functional glycosylation of α-dystroglycan [54, 55].

#### DAG1-CMD

The function of DAG1 is to link the extracellular matrix and the cytoskeleton in skeletal muscle. This disease typically was presented in early childhood and associated with intellectual disability without structural brain anomalies. Morpholino blocking of this gene shows that the fish model recapitulates muscle dystrophy, with significant loss of muscle integrity. Alterations in the structure of the sarcomere and sarcoplasmic reticulum were observed. Structures like fins and the tail are smaller and hooked in 2 dpf fish, which move in an uncoordinated manner [56].

#### Dowling-Degos disease (mutation in gene POFUT1)

Protein O-fucosyltransferase 1 add a fucose through an O-glycosidic linkage to a conserved serine or threonine residue found in the consensus sequence. Affected individuals develop a postpubertal reticulate hyperpigmentation that is progressive and disfiguring, and small hyperkeratotic dark brown papules that affect mainly the flexures and great skin folds. Changes in pigmentation are observed in zebrafish too, distribution of melanin in tail and body decreased significantly at 72hpf [56, 57].

#### Walker-Warburg syndrome (mutation in gene POMK)

Protein O-mannose Kinase mediates phosphorylation of an O-mannose. The most common phenotypes include brain anomalies, eye malformations, profound intellectual disability, and death typically occurring within the first years of life [58]. The zebrafish morpholino model recapitulates the majority of these phenotypes. For instance, zebrafish exhibit high mortality at 2 to 3 dpf, microcephaly, and delayed ocular development. Furthermore, muscular weakness is observed, along with reduced motility, impaired spontaneous movements, and inability to swim normally. Analysis of dystroglycans in muscle tissue shows a reduction in glycosylation [59].

#### Retinitis pigmentosa (mutation in gene POMGNT1)

Protein O-mannose N-acetylglucosaminyltransferase 1 catalyzes the addition of N-acetylglucosamine to O-linked mannose. This disease is characterized by ocular abnormalities that included a large list of pathologies. Also known as muscle-eye-brain disease due to the manifestation of the patients [60]. The zebrafish CRISPR/Cas9 model confirmed ocular abnormalities, demonstrating a reduction in O-mannosyl glycans and photoreceptor degeneration in the retina at 6 months post-fertilization (mpf) [59, 61].

#### POMT1-CMD

Protein O-mannosyltransferase 1 catalyzes the addition of O-linked mannose to α-dystroglycan, initiating the assembly of functional glycans as the protein is translated in the ER. In zebrafish, *pomt1* mutation at exon 19 induced by ENU has been studied. *pomt1* heterozygous juveniles exhibit reduced body lengths and a significantly thinner retinal and photoreceptor layer, with the latter showing a more pronounced thinning. Additionally, reduced swimming distance and velocity were observed, and these juveniles do not survive beyond the first 40 days post-fertilization [62]. In humans, mutations in *POMT1* can cause various congenital muscular dystrophy phenotypes, including brain and eye abnormalities [63].

#### Diastrophic dysplasia (mutation in gene SLC26A2)

Diastrophic dysplasia sulfate transporter participates in the proper sulfation of proteoglycans. Patients with this mutation typically present with steochondrodysplasia, characterized by clinical features such as dwarfism, spinal deformities, and specific joint abnormalities. This mutation is more common in the Finnish population [64]. The morpholino model for this gene focuses on the inner ear, showing malformations in otolith patterns, semicircular canal morphology, and lateral neuromast distributions. The fish model is insensitive to sound stimulation and exhibits imbalanced swimming behaviors [65].

### Zebrafish models for multiple glycosylation disorders

#### COG4-CDG (also known as Saul-Wilson syndrome)

Conserved Oligomeric Golgi Complex Subunit 4 participates in both N-glycosylation and O-glycosylation and is involved in Golgi-to-ER retrograde transport, ensuring proper glycan processing. COG4-CDG is a skeletal dysplasia characterized by short stature, distinctive craniofacial features, short fingers and toes. Other common symptoms include developmental delays in speech and motor skills, hearing loss, cataracts, and retinal dystrophy [66]. CRISPR-Cas9-generated zebrafish exhibit malformed inner ears with abnormally shaped semicircular canals, a reduced number of mechanosensory hair cells, and impaired auditory responses. Craniofacial defects, including a smaller jaw, underdeveloped inner ears, and stubby pectoral fins, have also been noted [67]. Further studies on *cog4* mutant zebrafish have identified additional abnormalities, such as shortened body length, gastrulation issues, defects in Meckel’s cartilage, cyclopia, and distinctive pectoral fin malformations [68]. These findings demonstrate the critical role of *COG4* in early development and its contribution to the phenotypes observed in Saul-Wilson syndrome.

#### GMPPB-CDG

GDP-mannose pyrophosphorylase B is an enzyme catalyzes the formation of GDP-mannose, which is essential for the glycan precursor assembly. Patients with mutations in *GMPPB* are classified under α-dystroglycanopathies. Patient symptoms include muscular defects, such as muscular dystrophy, isolated episodes of rhabdomyolysis, and neurological issues like epilepsy [69]. Two zebrafish morpholino models have been developed to study this condition, both showing similar results, including decreased locomotion, reduced movement distance, and slower movement speed. Researchers also observed structural muscle defects, with sparse and disordered muscle fibers [69]. Additionally, neuronal defects were identified, particularly in the reduced axonal length of CaP neurons (a type of spinal cord neuron) [70]. Both models effectively reproduce the phenotype observed in patients.

#### NANS-CDG

N-acetylneuraminate synthase converts N-acetylmannosamine-6-phosphate to N-acetylneuraminic acid-9-phosphate. The disorder is characterized by global developmental delay with infantile onset, intellectual disability, skeletal dysplasia, and short stature [71]. These phenotypes are also observed in a zebrafish morpholino model, where the fish exhibit developmental anomalies of the skeleton, microcephaly, and cartilage abnormalities in head structures. Treatment by supplementation with sialic acid has shown partial rescue of the skeletal phenotype [72].

#### SLC35C1-CDG

Solute Carrier Family 35 Member C1 is a transmembrane protein that function as nucleotide sugar transporters. Patients typically present in infancy or early childhood and are primarily characterized by a compromised immune system, short stature, cognitive impairment, and a rare blood type known as Bombay blood type. The zebrafish model called slytherin (*srn*) exhibits a significant neuronal phenotype, including malformation of the hindbrain, increased neurogenesis, cell death, and abnormal neuromuscular and central nervous system synaptic connectivity. Reduction of glycosylation was confirmed by lectin staining, which is strongly reduced in *srn* mutants. This model shows that protein fucosylation is dramatically reduced in the central nervous system and other tissues. The authors attempted to improve the phenotypes by supplementing with GDP-fucose, which rescues the slytherin phenotypes [73]. The supplementation treatment has also been tested in humans. Oral L-fucose supplementation showed biochemical and some clinical benefits, particularly in improving immunity and glycoprotein fucosylation [74].

#### TRAPPC11-CDG

Transport Protein Particle Complex 11 is involved in vesicle trafficking from endoplasmatic reticulum to cis-Golgi. This zebrafish model characterized by hepatic steatosis; as the name reflects: *foie grass* model. The analysis of the zebrafish model shows a fragmented and vesiculated secretory organelles in Golgi and endoplasmic reticulum at 5 dpf [75].

### Zebrafish models for other disorders

#### PIGK-CDG

Phosphatidylinositol Glycan Anchor Biosynthesis Class K mediates GPI anchoring in the endoplasmic reticulum. The disease is characterized by global developmental delay, intellectual disability, hypotonia, cerebellar ataxia, cerebellar atrophy, delayed motor skills, poor or absent speech, and epilepsy in most patients. Some patients also exhibit facial dysmorphism. Several of these symptoms are replicated in the zebrafish morpholino model, including craniofacial abnormalities such as reduced jaw size and decreased distances between some facial structures. Additionally, neuronal defects are observed, including neural cell death and the disappearance of axons of primary motor neurons, which explain the neuronal symptoms seen in patients [76].

## Conclusion

This review represents the first systematic approach to explore the use of zebrafish models in the study of Congenital Disorders of Glycosylation, addressing the key scientific question: *“What zebrafish models have been developed to study congenital disorders of glycosylation*,* and how do their findings compare to the clinical manifestations observed in human patients?“.*

This systematic review offers a comprehensive perspective on the topic and allows us to derive several key conclusions. Glycosylation genes are highly conserved across species, reinforcing the validity of the zebrafish as a model for studying Congenital Disorders of Glycosylation. Notably, the majority of zebrafish models focus on N-glycosylation disorders, indicating a predominant research interest in this area. The morpholino genetic editing technique remains the most widely employed in these models. Moreover, zebrafish models effectively replicate numerous clinical features observed in human CDG patients, providing valuable insights into the pathophysiology of these disorders.

In summary, zebrafish are an ideal model organism for CDG research due to their high genetic homology with humans, particularly in glycosylation pathways. Their ability to enable high-throughput screening and real-time observation of disease phenotypes offers valuable insights into the mechanisms by which specific gene mutations lead to the diverse and complex symptoms seen in CDG patients. Continued development and standardization of zebrafish models will further enhance their utility in understanding the underlying mechanisms of CDG and developing potential therapeutic strategies.

## Supplementary Information

Below is the link to the electronic supplementary material.

Supplementary Material 1

## Data Availability

All data generated or analysed during this study are included in this published article and its supplementary information files.

## Abbreviations

CDG: Congenital Disorders of Glycosylation
dpf: Days post-fertilization
ER: Endoplasmatic reticulum
hpf: Hours post-fertilization
IRDiRC: International Rare Diseases Research Consortium
PRISMA: Preferred Reporting Items for Systematic Reviews and Meta-Analyses
